# Supplementary material for: Brain criticality predicts individual levels of inter-areal synchronization in human electrophysiological data
Source: Nat Commun. 2023 Aug 7;14:4736. doi: 10.1038/s41467-023-40056-9 (PMC10406818; doi:10.1038/s41467-023-40056-9)
Supplement: Supplementary file 1 — Supplementary Information [file 41467_2023_40056_MOESM1_ESM.pdf]

ID	EZ Location	Drugs	Outcome (Engel score)
1	Right mesial frontal	Carbamazepine 600mg, Levetiracetam 1500mg	IB (8 years)
2	Right temporal insular	Carbamazepine 1200mg, Primidone 750 mg, Clonazepam 10mg	IA (37 months)
3	Right temporal	Carbamazepine 1200mg, Dilantin 450mg, Lacosamide 300mg, Clonazepam 10mg	No surgery
4	Left temporo-parietal	Phenobarbital 100mg, Topiramate 100mg, Levetiracetam 3000mg	IA (25 months)
5	Left temporal-insular	Oxcarbamazepine 600mg, Lacosamide 400mg	IA (15 months)
6	Right temporo-insular	Levetiracetam 1000mg, Lacosamide 350mg, Sertraline 50mg, Lorazepam 1mg	IIIA (32 months)
7	Left temporal- orbital	Carbamazepine 1200 mg, Levetiracetam 3500mg, Topiramate 200mg	IA (3 years)
8	Precuneus	Oxcarbamazepine 600mg, Lacosamide 400mg	No surgery
9	Functional epilepsy	Carbamazepine 1200mg, Levetiracetam 1500mg	No surgery
10	Right occipito-temporo-parietal	Lamotrigine 400mg, Levetiracetam 3000mg, Lacosamide 400mg	IA (3 years)
11	Right temporo-occipital	Phenytoin 200mg, Lacosamide 300mg	IA (6 months)
12	Left temporal	Topiramate 200mg, Carbamazepine 900mg	IA (12 months)
13	Right temporal anterior	Carbamazepine 1200mg	IIA (66 months)
14	Temporo-hip	Carbamazepine 1400mg, Levetiracetam 3000mg	IIA (6 months)
15	Left frontal anterior	Levetiracetam 2750mg, Carbamazepine 800mg, Primidone 750mg	IA (12 months)
16	Thermo-coagulation mult. sites	Oxcarbazepine 1800mg, Clobazam 20mg	IA (36 months)
17	Right fronto-temporo-insular	Carbamazepine 1000mg, Clobazam 20mg, Lomotrigine 200mg	IVA (24 months)
18	Left parieto-opercolo-insular	Carbamazepine 1200mg, Clobazam 40mg, Phenobarbital 75mg	IVA (12 months)
19	Right temporo-perisilvian	Phenobarbital 150mg, Lacosamide 400mg, Clobazam 10mg	IIC (38 months)
20	Right perisilvian-insular	Carbamazepine 800 mg, Lamotrigine 400mg	IVA (26 months)
21	Right temporo-parieto-occipital	Oxcarbazepine 1200mg, Phenobarbital 150mg, Valproate 1000mg	IA (35 months)
22	--	Carbamazepine 700mg	No surgery
23	Left temporal antero-mesial	Carbamazepine 1200mg, Levetiracetam 750mg	IA (61 months)
24	Right fronto-centro-insular	Carbamazepine 800mg, Lacosamide 800mg, Zonisamide 250mg	IIA (38 months)
25	Left temporal	Levetiracetam 1750mg, Lacosamide 400mg, Valproate1000mg	IA (24 months)
26	Right parietal	Levetiracetam 3000mg, CBZ 1000mg, Lacosamide 500mg	IA (24 months)
27	Thermo-coagulation mult. sites	Carbamazepine 1200mg, Phenobarbital 100mg	IA (24 months)
28	Right frontal	Valproate 800mg, Clobazam 10mg	IIA (36 months)
29	Right fronto-mesial	Carbamazepine 800mg, Levetiracetam 3000mg, Nitrazepam 1.5mg	IIIA (13 months)
30	Right fronto-central	Lamotrigine 400mg, Levetiracetam 2000mg	IA (24 months)
31	Right frontal	Carbamazepine 600mg, Rufinamide 1500mg	IVA (13 months)
32	Right frontal	Carbamazepine 1200mg, Zonisamide 400mg, Phenobarbital 1000mg	IC (24 months)
33	--	Carbamazepine 300mg	No surgery
34	--	Levetiracetam 1500 mg, Clobazam 5mg	No surgery
35	Right temporal antero-mesial	Oxcarbazepine 2000mg, Phenobarbital 150mg	IIA (36 months)
36	--	Carbamazepine 16000mg, Levetiracetam 4000mg	No surgery
37	--	Levetiracetam 3000mg	No surgery
38	Right orbito-temporal	Zonisamide 400mg, Levetiracetam 750mg, CBZ 1400mg	IA (62 months)
39	--	Lacosamide 500mg, Valproate 1000mg, Zonisamide 200mg	No surgery
40	Thermo-coagulation mult. sites	Carbamazepine 1000mg, Levetiracetam 2500mg	IA (12 months)
41	Thermo-coagulation mult. sites	Levetiracetam 2000mg, Lacosamide 600mg	IIA (6 months)
42	Left occipital	Carbamazepine 1200mg, Levetiracetam 1500mg, Lacosamide 300mg	IB (49 months)
43	Right temporal	Topiramate 300mg, Oxcarbamazepine 1200mg	IIA (50 months)
44	--	Carbamazepine 800mg, Lamotrigine 200mg	No surgery
45	--	Carbamazepine 1200mg, Levetiracetam 3000mg, Lacosamide 150mg, Clobazam 20mg	No surgery
46	Left cingulum	Oxcarbamazepine 1800mg, Topiramate 200mg, Levetiracetam 3000mg, Clobazam 10mg	IA (16 months)
48	Thermo-coagulation mult. sites	Topiramate 200mg, Lamotrigine 200mg	IA (5 years)
49	Right temporal antero-mesial	Lacosamide 500mg	IB (36 months)
50	Left parieto-temporal	Carbamazepine 900mg	IA (6 months)
51	Thermo-coagulation mult. sites	Carbamazepine 900mg, Levetiracetam 3000mg	IVA (12 months)
52	Left frontal	Carbamazepine 1200mg, Lamotrigine 200mg, Clobazam 20mg	IA (5 years)
53	Left frontal	Levetiracetam 1250mg, Oxcarbamazepine 1200mg	IA (4 years)
54	Thermo-coagulation mult. sites	Levetiracetam 3000mg, Lacosamide 400mg	IA (2 years)
55	Right temporo-occipital	Lamotrigine 600mg, Levetiracetam 2000mg	IA (2 years)
56	Right temporal	Carbamazepine 1400mg, Levetiracetam 3000mg, Clobazam 10mg	IA (31 months)
57	Left opercolo-insular	Carbamazepine 800mg	IIIA (34 months)
58	Right temporo-frontal	Lamotrigine 600mg, Clobazam 20mg, Phenytoin 500mg	IA (13 months)
60	Right temporal	Lamotrigine 400mg, Topiramate 400mg	IA (24 months)
62	Left temporal	Oxcarbamazepine 1500mg, Clobazam 20mg, Levetiracetam 2500mg	IA (36 months)
63	Right temporo-mesial	Topiramate 75mg, CBZ 1500mg	IA (12 months)
64	Nodular heterotopia	Carbamazepine 1000mg, Levetiracetam 500mg, Clobazam 20mg	IVA (12 months)
65	Left temporo-perisilvian	Carbamazepine 1200mg, Lamotrigine 550mg	IIIA (12 months)
66	Left temporal antero-mesial	Clobazam 20mg, Phenobarbital 45mg	IIA (55 months)
67	Right temporo-occipital	Carbamazepine 800mg, Levetiracetam 3000mg, Phenobarbital 125mg	IA (24 months)

Supplementary Table 1.

Clinical information for the analyzed SEEG cohort. EZ location refers to the identified brain location of the epileptogenic zone, with dashed entries indicating that no single focal location was identified. Drugs are reported by the scientific name of their principal active component. Dosage is expressed in milligrams and refers to the morning dosage measured at the day of the recording. Outcome is expressed as Engel scores and in parenthesis we report the time between surgery and the clinical follow-up when the outcome was assessed.

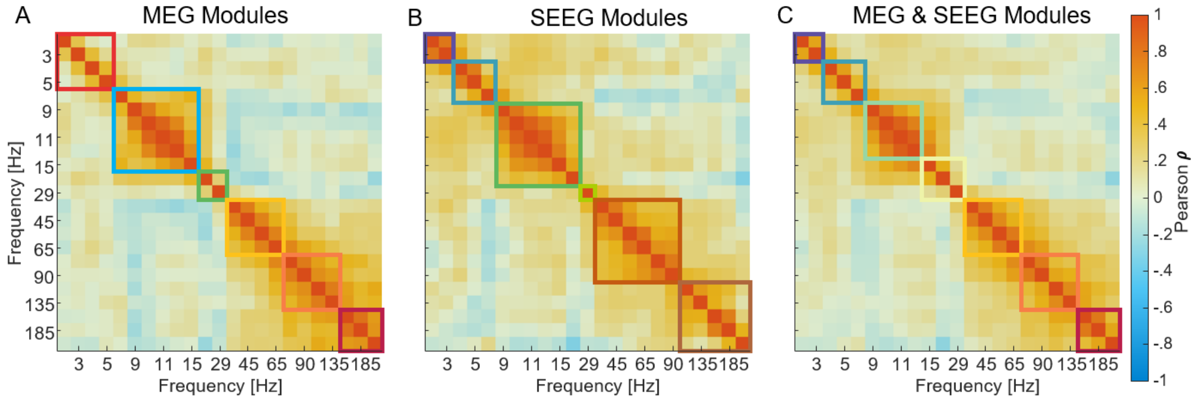

Supplementary Figure 1.

A. Spatial similarity analysis between frequencies using Louvain clustering (Blondel et al., 2008). Pearson coefficients of DFA exponents were estimated between frequency pairs across parcels or regions and were used as a directed weighted matrix in a multi-iterative Louvain community detection algorithm with the resolution parameter $\gamma = 1.5$ to identify modules (note that $0.5 \leq \gamma \leq 1$ leads to only 2 modules, dividing low and high frequency bands at 32 Hz). **B**. Same as in A. but for SEEG contacts. **C.** Same for combined MEG and SEEG data.

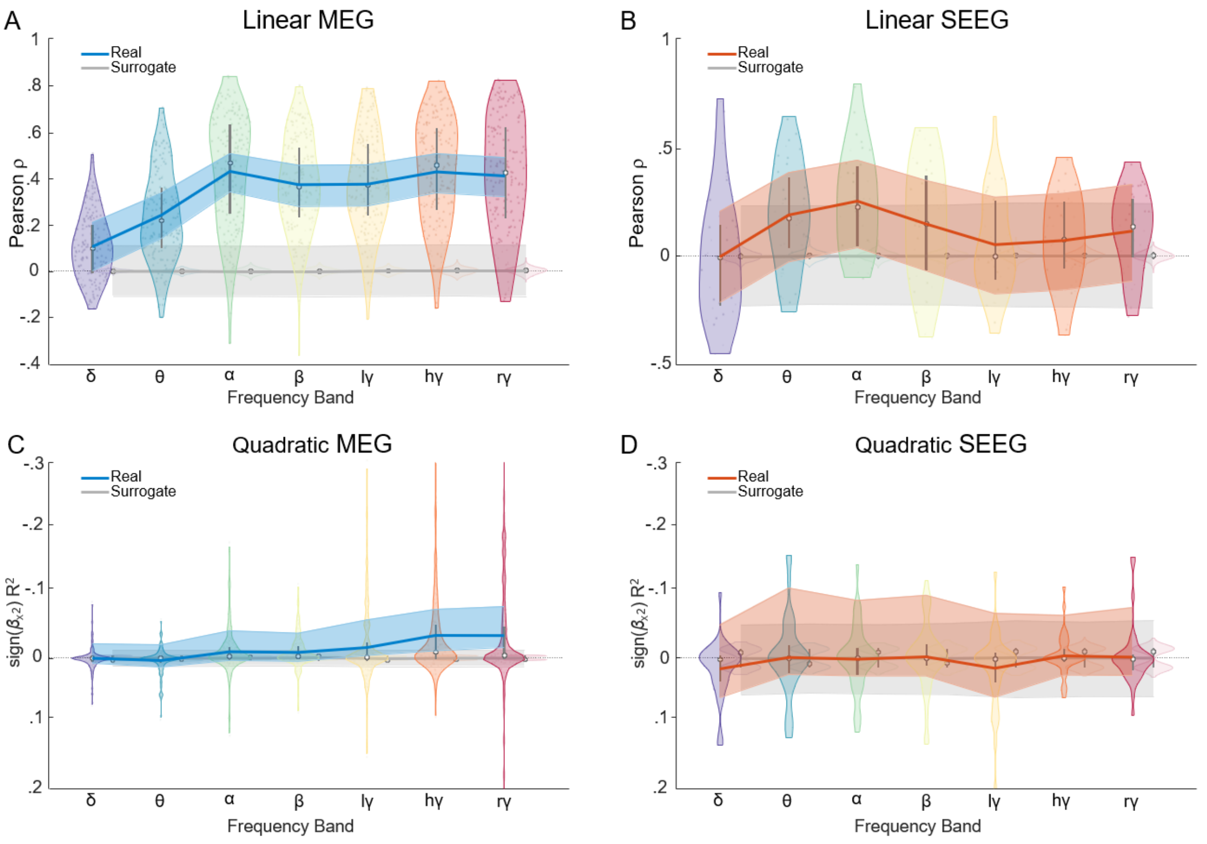

Supplementary Figure 2.

Linear and quadratic correlations of node strength and DFA exponents. **A.** Means and distributions of linear Pearson correlation coefficients for different frequency bands in MEG parcels. Means of the real correlations are given in blue, with shaded areas indicating 95% confidence intervals. Surrogate mean correlations obtained by case-resampling in grey, with the shaded areas indicating the 2.5-97.5th percentiles of the surrogate coefficients distribution. Violin plots show correlation coefficients distributions, with the median indicated by square and quartiles by notch indicators. **B.** Same as in A, for SEEG contacts. **C,D.** Same as above for partial quadratic (linear component removed) correlations multiplied with the sign of the quadratic coefficient (notice the y-axis has negative values on top).

Frequency bands: δ: 2−4 Hz; θ: 4−8 Hz; α: 8−14 Hz; β: 15−29 Hz; lγ 30−70 Hz; hγ: 71−135 Hz; rγ: 165−225 Hz

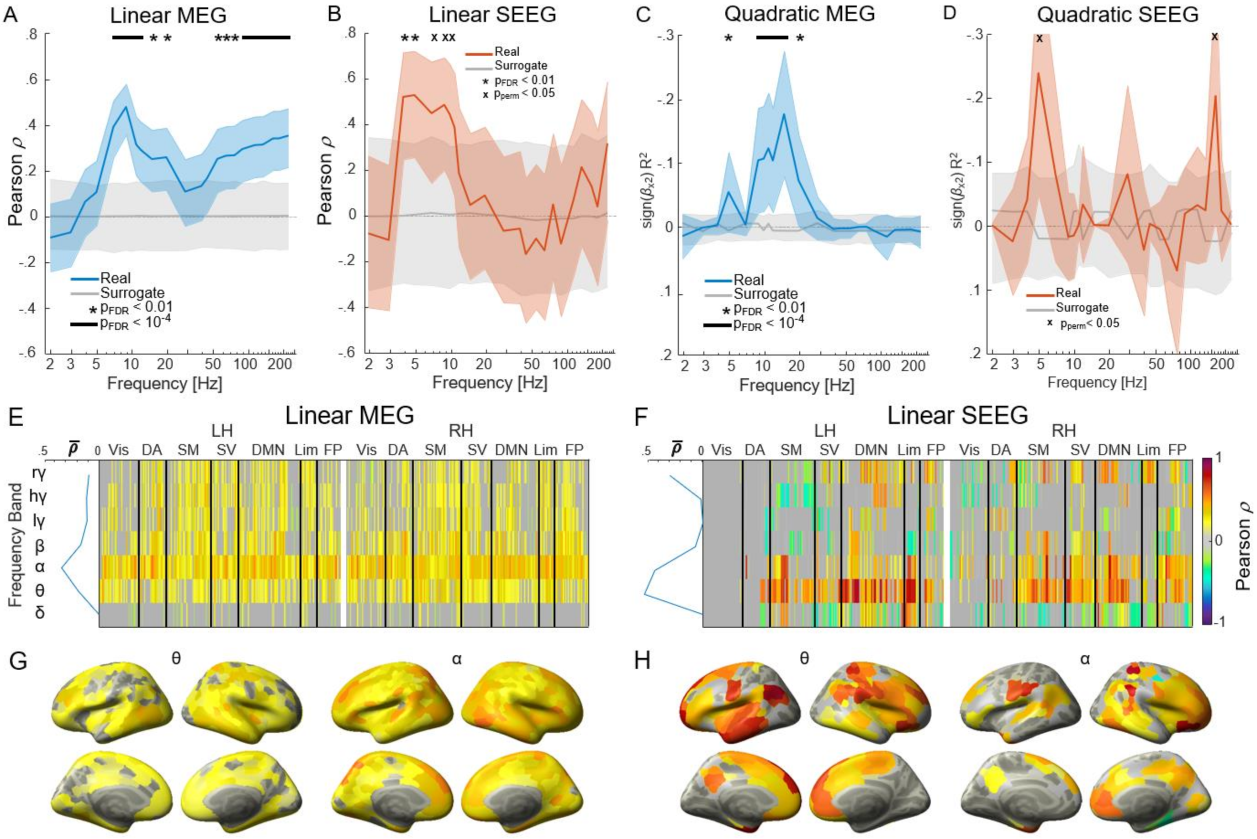

Supplementary Figure 3.

A. Mean linear correlations (Pearson's ρ) of graph strength and mean detrended fluctuation analysis (DFA) exponents using PLV for MEG and **B**. Mean weighted phase-lag index (wPLI) for SEEG (the opposite connectivity metrics as were used in main figure). Mean correlations with 95% confidence intervals in blue or red, respectively, with grey area indicating 2.5-97.5th percentiles surrogate distribution. Asterisks at the top indicate $p_{FDR} < 0.01$ and the black line $p_{FDR} < 10^{-4}$ (case-resampling permutation test, 2-sided, significant after FDR correction with Benjamini-Hochberg). **C.** Mean partial quadratic correlations with 95% CI for MEG and **D.** for SEEG. **E.** Mean correlation values in brain across parcels for MEG and **F.** for SEEG. **G.** Cortical correlations topographies of mean node strength and DFA exponents for the θ (4 – 8 Hz) and α (8 – 14 Hz) frequency bands. **H.** Same as in G for SEEG.

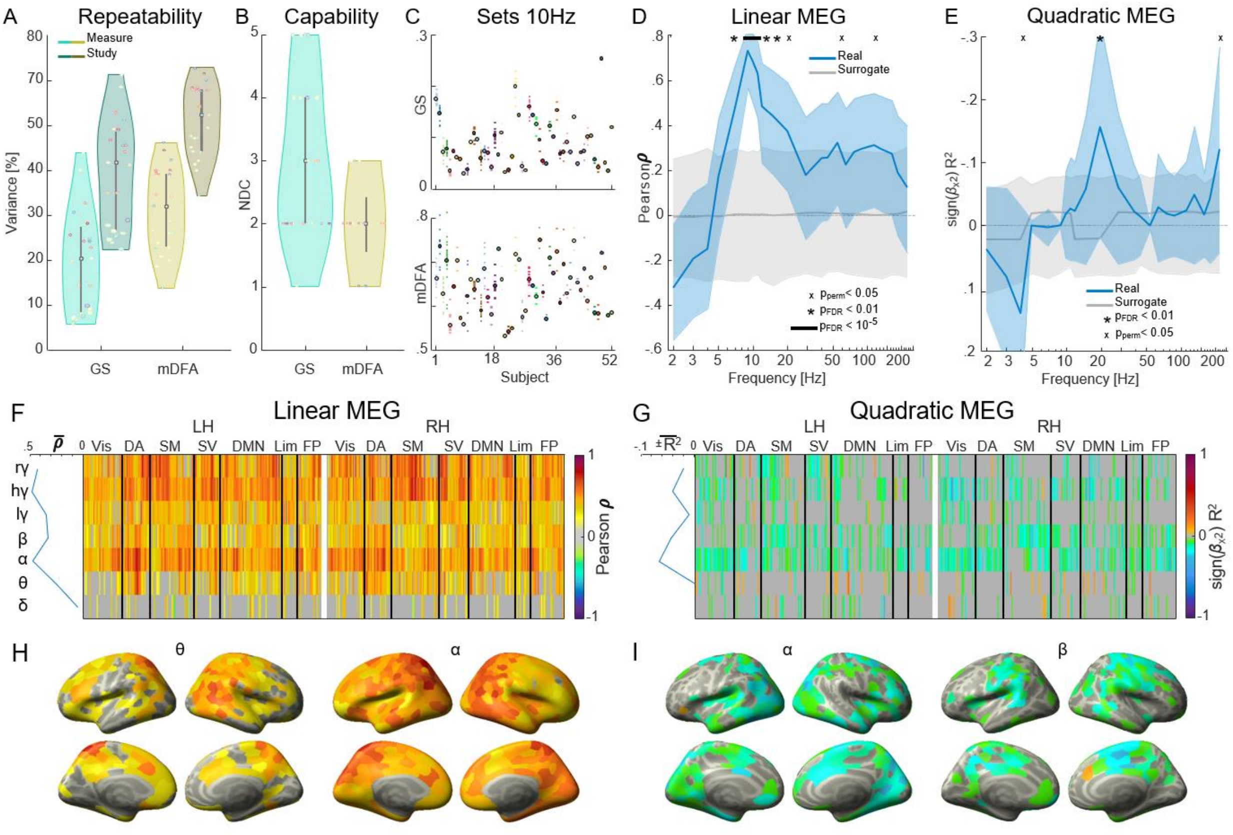

Supplementary Figure 4.

Repeatability of measures. **A.** Gauge Repeatability (ANOVA, Burdick et al., 2005) of MEG weighted phase-lag index (wPLI) graph strength (GS) and detrended fluctuation analysis (DFA) exponents (N = 192 recordings, bars denote median and 25th and 75th percentile). Violin plots show repeatability distributions of colour-coded frequencies as percentage of contributing variance, with median and quartiles notches. Darker violin plots show the study-wide variance. **B.** Single frequency distributions of the capability of the metrics as Number of Distinct Categories (NDC), presentation as in a. Larger points in the density violins are for frequencies with NDC > 3. **C.** Example MEG GS (top) and mean DFA (bottom) for 10 Hz across session for each subject (N = 52). The subject means, indicated with a bigger circle, were used in the subsequent analyses of this figure. **D.** Mean linear Pearson correlation coefficients with 95% CI of subject-averaged MEG GS vs. DFA across frequencies with the 2.5-97.5th percentiles surrogates in grey. 'x's indicate values beyond the 95% distribution of the surrogate correlations (2-sided test); asterisks at the top indicate $p_{FDR} < 0.01$ (significant after correction with Benjamini-Hochberg); the black line $p_{FDR} <$ 10-4. **E.** Mean partial quadratic correlations of subject average MEG GS and DFA with 95% CI given as R^2 times the sign of the quadratic β. **F.** Left and right hemisphere parcel-by-frequency-band matrices of linear correlations of subject-averaged MEG NS and DFA exponents. Blue lines on left indicate mean correlation per frequency band. **G**. Same as in F for partial quadratic correlations times the sign of the quadratic coefficient. **H**. Cortical correlations topographies of subject average MEG NS and DFA for the θ and α frequency bands. **I.** Same as in H for partial quadratic with the quadratic sign for the α and β frequency bands.

Frequency bands: δ: 2−4 Hz; θ: 4−8 Hz; α: 8−14 Hz; β: 15−29 Hz; lγ 30−70 Hz; hγ: 71−135 Hz; rγ: 165−225 Hz

Functional subsystems: Vis: Visual, DA: Dorsal Attention, SM: Somatomotor, SV: Salience and Ventral Attention, DMN: Default Mode Network, Lim: Limbic, FP: Frontoparietal.

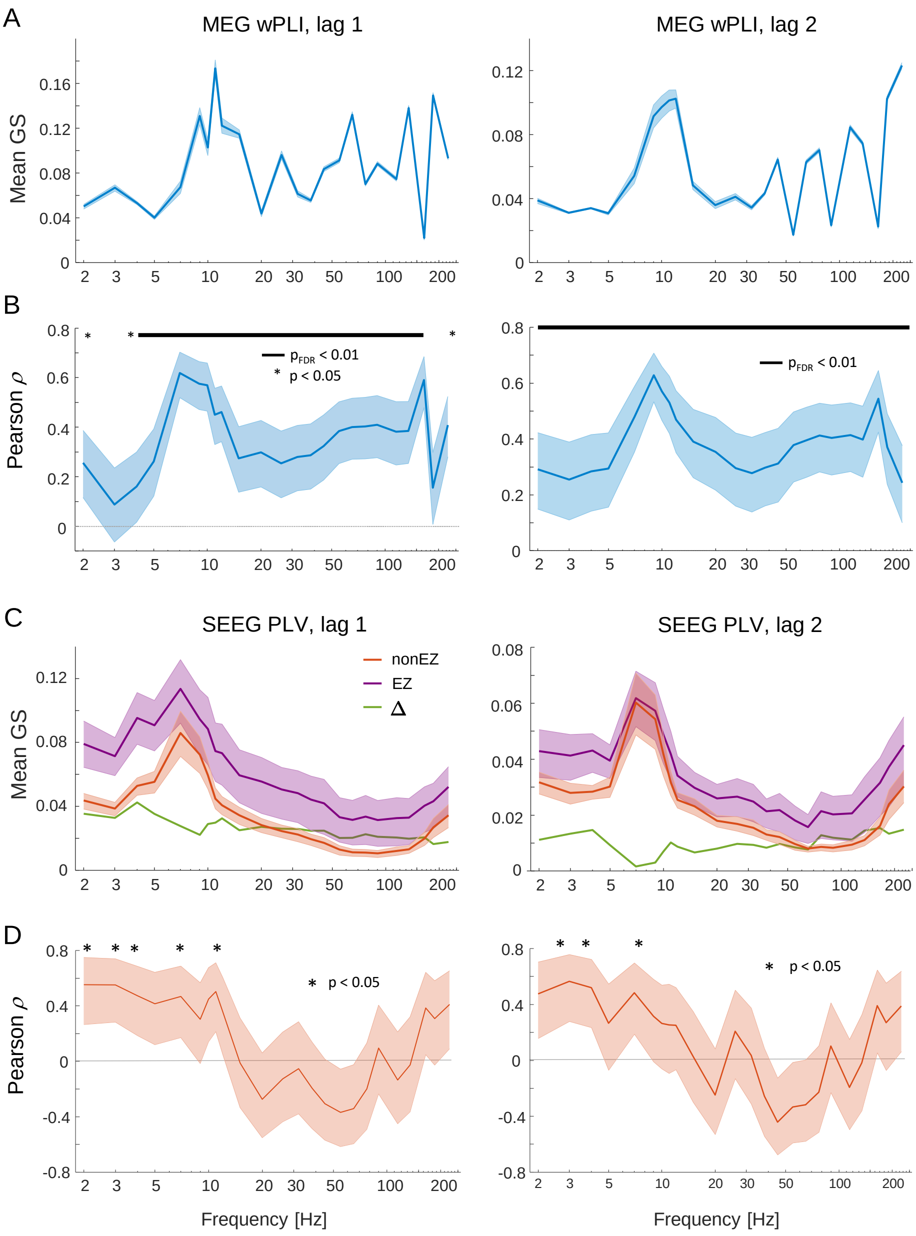

Supplementary Figure 5.

A. Mean graph strength (GS) of phase synchronization (assessed with the weighted phase-lag index, wPLI) in MEG data with lags of 1 and 2 cycles added between the two time series. **B.** Mean linear correlations (Pearson's ρ, with 95% CI) between wPLI and detrended fluctuation analysis (DFA) exponents in MEG data at lags 1 and 2. **C.** Mean phase synchronization (assessed with phase-locking value, PLV) in SEEG data with lags 1 and 2 for non-EZ (orange) and EZ (purple) regions and the difference between them (green). **D.** Linear correlations (Pearson's ρ) between PLV and DFA exponents for non-EZ regions.

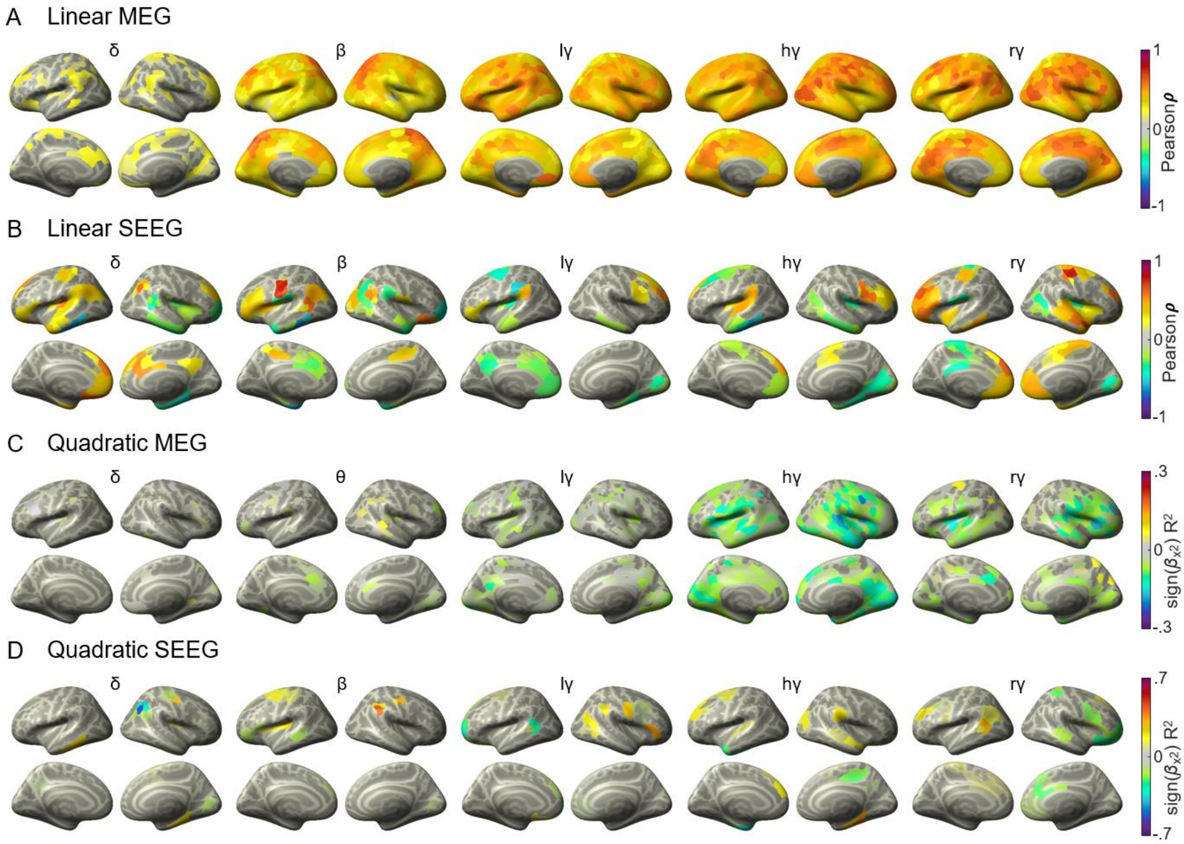

Supplementary Figure 6.

A-D. Cortical topographies of linear and quadratic correlations of phase synchronization node strength and detrended fluctuation analysis (DFA) exponents for all the frequency bands not shown in Figure 3.

Frequency bands: δ: 2−4 Hz; θ: 4−8 Hz; α: 8−14 Hz; β: 15−29 Hz; lγ 30−70 Hz; hγ: 71−135 Hz; rγ: 165−225 Hz

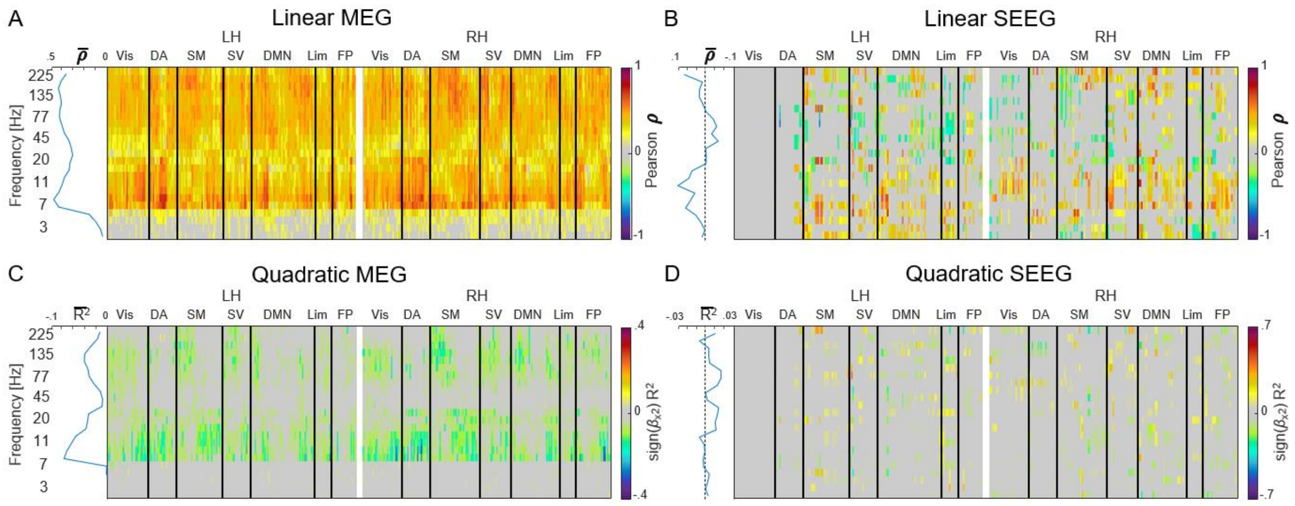

Supplementary Figure 7.

Linear and quadratic correlations of phase synchronization node strength and detrended fluctuation analysis (DFA) exponents for all parcels and single frequencies (compare main Figure 3 where these are shown for frequency bands). **A.** Left and right hemisphere parcel-by-frequency-matrices of linear correlations of MEG node strength and DFA. Non-significant correlations are masked. On the left side, blue lines represent the average (across significant parcels) correlation values per frequency. **B.** Same as in A, for SEEG. **C-D.** Same as above for partial quadratic (linear component removed) correlations multiplied with the sign of the quadratic coefficient.

Frequency bands: δ: 2−4 Hz; θ: 4−8 Hz; α: 8−14 Hz; β: 15−29 Hz; lγ 30−70 Hz; hγ: 71−135 Hz; rγ: 165−225 Hz

Functional subsystems: Vis: Visual, DA: Dorsal Attention, SM: Somatomotor, SV: Salience and Ventral Attention, DMN: Default Mode Network, Lim: Limbic, FP: Frontoparietal.

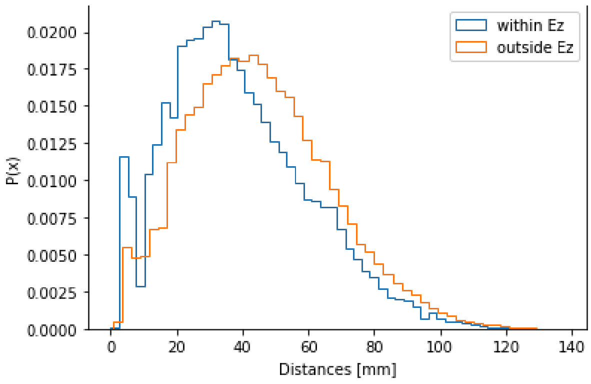

Supplementary Figure 8.

Distribution of pairwise distances between contacts for pairs either both within or both outside epileptic zone (EZ).
