# Supplementary material for: Brain criticality predicts individual levels of inter-areal synchronization in human electrophysiological data
Source: Nat Commun. 2023 Aug 7;14:4736. doi: 10.1038/s41467-023-40056-9 (PMC10406818; doi:10.1038/s41467-023-40056-9)
Supplement: Supplementary file 3 — Reporting Summary [file 41467_2023_40056_MOESM3_ESM.pdf]

nature portfolio

Corresponding author(s): Satu Palva

Last updated by author(s): Jun 22, 2023

Reporting Summary

Nature Portfolio wishes to improve the reproducibility of the work that we publish. This form provides structure for consistency and transparency in reporting. For further information on Nature Portfolio policies, see our Editorial Policies and the Editorial Policy Checklist.

Statistics

For all statistical analyses, confirm that the following items are present in the figure legend, table legend, main text, or Methods section.

n/a	Confirmed	
[ ]	[x]	The exact sample size (n) for each experimental group/condition, given as a discrete number and unit of measurement
[ ]	[x]	A statement on whether measurements were taken from distinct samples or whether the same sample was measured repeatedly
[ ]	[x]	The statistical test(s) used AND whether they are one- or two-sided<br>*Only common tests should be described solely by name; describe more complex techniques in the Methods section.*
[x]	[ ]	A description of all covariates tested
[ ]	[x]	A description of any assumptions or corrections, such as tests of normality and adjustment for multiple comparisons
[ ]	[x]	A full description of the statistical parameters including central tendency (e.g. means) or other basic estimates (e.g. regression coefficient) AND variation (e.g. standard deviation) or associated estimates of uncertainty (e.g. confidence intervals)
[ ]	[x]	For null hypothesis testing, the test statistic (e.g. F, t, r) with confidence intervals, effect sizes, degrees of freedom and P value noted<br>*Give P values as exact values whenever suitable.*
[x]	[ ]	For Bayesian analysis, information on the choice of priors and Markov chain Monte Carlo settings
[x]	[ ]	For hierarchical and complex designs, identification of the appropriate level for tests and full reporting of outcomes
[x]	[ ]	Estimates of effect sizes (e.g. Cohen's d, Pearson's r), indicating how they were calculated

Our web collection on statistics for biologists contains articles on many of the points above.

Software and code

Policy information about availability of computer code

Data collection	MEG acquisition software (Elekta-Neuromag/MEGIN, Helsinki, Finland) was used in the recording of MEG data. MRI acquisition hard- and software by Siemens (Munich, Germany) was used in MRI recordings at Helsinki University Central Hospital.<br><br>SEEG data were collected using Nihon Kohden (Tokio, Japan) software (v1100A, 1200A). Philips MRI acquisition software (Amsterdam, Netherlands) was used in MRI recordings at Niguarda Hospital. CT data was recorded with acquisition software by Medtronic (Minneapolis, USA).
Data analysis	Freesurfer (v.5, https://surfer.nmr.mgh.harvard.edu/) was used for processing MRI data. Maxfilter (v.2, Elekta-Neuromag/MEGIN, Helsinki, Finland) software and MNE software package (v.0.23, www.mne.tools), was used for preprocessing MEG data. Data analysis was carried out with custom code written in Python (v.3.9, https://www.anaconda.com/) and Matlab (v.2018, www.mathworks.com) using the FieldTrip toolbox (v.2012, https://www.fieldtriptoolbox.org/). Our custom code is available under: https://github.com/palvalab/DFA_Synch.

For manuscripts utilizing custom algorithms or software that are central to the research but not yet described in published literature, software must be made available to editors and reviewers. We strongly encourage code deposition in a community repository (e.g. GitHub). See the Nature Portfolio guidelines for submitting code & software for further information.

Data

Policy information about availability of data

All manuscripts must include a data availability statement. This statement should provide the following information, where applicable:

- Accession codes, unique identifiers, or web links for publicly available datasets
- A description of any restrictions on data availability
- For clinical datasets or third party data, please ensure that the statement adheres to our policy

A minimal dataset containing phase synchronization matrices and DFA exponents, as well as supporting data, is available at DataDryad repository (https://doi.org/10.5061/dryad.vdncjsxzn).

Human research participants

Policy information about studies involving human research participants and Sex and Gender in Research.

Reporting on sex and gender	MEG data was recorded from 52 participants of which 27 were of male sex, and SEEG data from 68 participants of which 38 were of male sex.
Population characteristics	MEG data was recorded from 52 healthy participants (age: 31 ± 9.2 years) in Helsinki, Finland. SEEG data was recorded in Milan, Italy, from 68 drug-resistant focal epileptic patients (age: 30 ± 9.4 years).
Recruitment	MEG participants were recruited among university employees and students and their close contacts. Therefore there may exist an educational bias. However, as this study is basic research, we do not assume that such a bias would have any qualitative effect on results.<br><br>SEEG participants were recruited at the Niguarda Hospital of Milan, Italy, progressively without restrictions. Individuals that had undergone previous neurosurgical interventions or had significant brain injuries (e.g. tumors) were excluded, as described in Arnulfo et al., 2020, Nature Communications. We are not aware of any potential bias in this cohort.
Ethics oversight	The study protocol for MEG and MRI data was approved by the Coordinating Ethical Committee of Helsinki University Central Hospital (ID 290/13/03/2013), written informed consent was obtained from each participant prior to the experiment, and all research was carried out according to the Declaration of Helsinki.<br>The ethical committee of the Niguarda Hospital, Milan, approved the SEEG study (ID 939) which was performed according to the Declaration of Helsinki.

Note that full information on the approval of the study protocol must also be provided in the manuscript.

Field-specific reporting

Please select the one below that is the best fit for your research. If you are not sure, read the appropriate sections before making your selection.

☒ Life sciences ☐ Behavioural & social sciences ☐ Ecological, evolutionary & environmental sciences

For a reference copy of the document with all sections, see nature.com/documents/nr-reporting-summary-flat.pdf

Life sciences study design

All studies must disclose on these points even when the disclosure is negative.

Sample size	We estimated, using standard sample size estimation methods, that in order to discover correlation coefficients of 0.4 with alpha = 0.05 and Type II Error rate of 20%, at least 47 participants were needed. Such coefficients represent at least medium-to-large effect sizes.
Data exclusions	No participants were excluded from MEG analysis. For each participant, parcels with low reconstruction accuracy and parcel pairs with high artificial synchrony were excluded (14.9 ± 0.2% of parcels and 14.1 ± 0.1% of edges rejected on average per set).<br><br>From SEEG data, 3 participants were excluded in which more than 50% of contacts were defective. From others, contacts demonstrating non-physiological activity were excluded. Epileptogenic zone (EZ) and seizure propagation networks were identified by clinical expert analyses of the clinical data and quantitative diagnostic imaging data including SEEG traces, which were confirmed later by another expert.<br>Analyses were carried out in two ways: once utilizing only non-EZ contacts and excluding 8 subjects with the highest number of EZ contacts, and once utilizing only EZ contacts and excluding 8 subjects with the lowest number of EZ contacts. Thus, both types of analysis included 57 subjects.
Replication	We used the Gauge Repeatability method (Burdick et al., 2005) to confirm that both individual GS and DFA values and their correlations had significant retest-reliability and -capacity. This test of replicability was carried out once and was successful.

Randomization	No allocation of participants into experimental groups took place.
Blinding	No allocation of participants into experimental groups took place.

Reporting for specific materials, systems and methods

We require information from authors about some types of materials, experimental systems and methods used in many studies. Here, indicate whether each material, system or method listed is relevant to your study. If you are not sure if a list item applies to your research, read the appropriate section before selecting a response.

Materials & experimental systems

n/a	Involved in the study	
☒	☐	Antibodies
☒	☐	Eukaryotic cell lines
☒	☐	Palaeontology and archaeology
☒	☐	Animals and other organisms
☒	☐	Clinical data
☒	☐	Dual use research of concern

Methods

n/a	Involved in the study	
☒	☐	ChIP-seq
☒	☐	Flow cytometry
☒	☐	MRI-based neuroimaging
